# Establishing Waist-to-Height Ratio Standards from Criterion-Referenced BMI Using ROC Curves in Low-Income Children

**DOI:** 10.1155/2016/2740538

**Published:** 2016-11-03

**Authors:** Ryan D. Burns, Timothy A. Brusseau, Yi Fang, You Fu, James C. Hannon

**Affiliations:** ^1^Department of Health, Kinesiology, and Recreation, University of Utah, 250 S. 1850 E., HPER North, RM 241, Salt Lake City, UT 84112, USA; ^2^School of Community Health Sciences, University of Nevada, Reno, 1664 North Virginia Street, Reno, NV 89557, USA; ^3^College of Physical Activity and Sport Sciences, West Virginia University, P.O. Box 6116, 375 Birch St., Morgantown, WV 26505-6116, USA

## Abstract

The purpose of this study was to establish health-related waist-to-height ratio (WHtR) cut-points associating with FITNESSGRAM's body mass index (BMI) criterion-referenced standards in low-income children. A secondary aim was to examine the classification agreement between the derived WHtR cut-points and various cardiometabolic blood markers using current recommendations. Participants were 219 children from low-income schools (mean age = 10.5 ± 0.6 years). Waist circumference, height, weight, and cardiometabolic blood markers were collected in a fasting state before school hours. Receiver operating characteristic (ROC) curves were used to determine WHtR cut-points that associated with a child meeting FITNESSGRAM's age- and sex-specific criterion-referenced standards for BMI. The derived WHtR cut-point was 0.50 (AUC = 0.89, *p* < 0.001; sensitivity = 0.86, specificity = 0.82, and accuracy = 84.3%). Classification agreement using the derived WHtR cut-point with various blood marker standards was statistically significant but considered weak to fair (kappa 0.14–0.34, agreement = 59%–67%, and *p* < 0.01). The WHtR cut-point of 0.50 can be used with strong accuracy to distinguish low-income children who met FITNESSGRAM's criterion-referenced standards for body composition; however, the evidence was weaker for its use in distinguishing low-income children meeting specific cardiometabolic blood marker recommendations.

## 1. Introduction

Optimizing health-related fitness (HRF) is an effective strategy for attenuating cardiometabolic disease health risk in the pediatric population [1, 2]. HRF consists of five components including body composition, cardiorespiratory endurance, muscular strength and endurance, and flexibility [3]. Of these five components, body composition and cardiorespiratory endurance have the strongest links to health outcomes in children and adolescents [4, 5]. Therefore, optimizing body composition and cardiorespiratory endurance may decrease the incidence of cardiometabolic disease risk factors [6]. This may especially be important in low-income children where the prevalence of obesity and unfavorable obesity-related cardiometabolic disease risk factors is significantly greater compared to higher socioeconomic status pediatric populations [7, 8].

Currently in the USA, HRF is assessed using the FITNESSGRAM, the national fitness test battery (http://www.fitnessgram.net). For body composition, FITNESSGRAM recommends the use of percent of body fat or body mass index (BMI) [9–11]. Because of the logistic limitations of acquiring estimated percent of body fat from sum of skinfolds assessment in physical education settings, BMI is often used because of its ease of administration and calculation. The BMI standards are currently age- and sex-specific when employing the FITNESSGRAM [3]. Instead of providing a child with an absolute BMI score, children are classified into Healthy Fitness Zones, which gives the child personalized messages based on their current estimated body composition [12]. The two primary Healthy Fitness Zones include the Healthy Fitness Zone (HFZ), a zone where a child is given a message stating that he or she is at a level of good health, and the Needs Improvement (NI), a zone that gives the message that a child should strive to improve their BMI to attenuate health risk. The current version of FITNESSGRAM (v.10) states that the cut-points for BMI are adjusted to align with the age- and sex-specific Centers for Disease Control and Prevention BMI percentiles, using the 83rd and 92nd percentiles for boys and the 80th and 90th percentiles for girls. These cut-points have been validated using a metabolic syndrome criterion in a large sample of adolescents aged 12–19 years from the National Health and Nutrition Examination Survey [13].

Despite the benefits of BMI in field settings, specifically in physical education, the index contains inherent limitations. The original development for BMI was for population surveillance, to monitor body composition within large groups of people [14]. However, it is currently widely used for individual body composition assessment [15]. Its use at the individual level has been under scrutiny because of its inability to distinguish between fat mass and fat-free mass [16]. BMI also does not specify where fat is distributed on a person's body, as visceral adipose deposits have been shown to pose more of a health risk compared to subcutaneous deposits [17].

An increasingly popular alternative to BMI is waist-to-height ratio (WHtR) [18]. WHtR is simply an individual waist circumference divided by their height. Some studies have shown that this index is better at distinguishing children and adolescents with unfavorable cardiometabolic risk factors than BMI [19]. However, the discordance in BMI and WHtR estimations of individual body composition may be more evident after the commencement of puberty in both girls and boys, when hormonal changes elicit different fat and fat-free mass distributions and deposit rates on the body [20]. Despite this, the use of WHtR in younger children still may have utility, especially to monitor central adiposity. Indeed, in the low-income pediatric population, central (or visceral) adiposity has been shown to be more prevalent compared to children of a higher socioeconomic status [20, 21].

No study to date has developed WHtR cut-points associating current FITNESSGRAM standards for body composition. Also, examining the agreement of the derived WHtR cut-point with cardiometabolic blood marker recommendations will give evidence for the strength of its utility in clinical settings for identifying low-income children at risk for early onset cardiometabolic disease. Therefore, the purpose of this study was to derive a WHtR cut-point associating with meeting FITNESSGRAM's age- and sex-specific criterion-referenced standards for BMI in low-income children from the USA. A secondary aim was to examine the classification agreement between children meeting the derived WHtR cut-point with meeting standards for various cardiometabolic blood markers using recommendations from the National, Heart, Lung, and Blood Institute.

## 2. Material and Methods

### 2.1. Participants

Participants were a nonprobability convenience sample of 219 children from five low-income schools from the Mountain West region of the US (mean age = 10.5 ± 0.6 years; 126 girls and 93 boys). Children were recruited from the fourth through the sixth grades and were primarily of a Hispanic ethnic background (210/219, 95.8%). Approximately 91%–96% of the children at each school were from low-income families. Written assent was obtained from each child and written consent was obtained from each child's parent or guardian prior to data collection. There were no exclusion criteria given for recruitment of the children and all children were in good physical condition for physical assessment. The University Institutional Review Board approved the protocols employed in this study.

### 2.2. Measures

#### 2.2.1. Body Mass Index and Waist-to-Height Ratio

Height was measured to the nearest 0.5 centimeter using a portable stadiometer (SECA 213; Hanover, MD, USA). With shoes off, weight was measured to the nearest 0.1 kg using a portable medical scale (BD-590; Tokyo, Japan). BMI was calculated taking each child's weight (in kg) divided by square of height (in meters). Waist circumference was measured in a private screening area where three abdominal circumference measurements were taken at the level of the superior border of the iliac crest on the participant's right side using a standard measuring tape. All measurements were estimated to the nearest 0.5-centimeter with the average of the three measurements used for data analysis. WHtR was calculated taking the child's waist circumference in centimeters divided by their height in centimeters. The anthropometric measures (i.e., BMI and waist circumference) were collected by a trained graduate research assistant to maintain testing consistency and were collected in accordance with the American College of Sports Medicine guidelines.

#### 2.2.2. Cardiometabolic Blood Markers

Each child's cardiometabolic biomarkers were collected using the Cholestech LDX system (Alere Inc., Waltham, MA, USA). Individual blood markers included total cholesterol, LDL cholesterol, HDL cholesterol, triglycerides, and blood glucose. A capillary blood sample was collected between the hours of 6 am and 8 am before the start of the school day. All blood samples were collected in a fasting state, verbally verified by both the child and the child's parent or guardian. Blood samples were collected using a finger stick on each child's right index finger using a 40 *μ*L capillary tube and injected into a Lipid Profile-Glucose Cassette (Alere Inc., Waltham, MA, USA) to be subsequently analyzed. The puncture site was cleaned and bandaged and all materials were properly disposed of in a biohazard container.

Blood pressure was measured using an electronic blood pressure device (CONTEC08A, Contec Medical Systems Co., Qinhuangdao, China). Systolic blood pressure and diastolic blood pressure measurements were taken on each child's right arm with the right arm rested and elevated at heart level and both feet flat on the ground. Blood pressure measurements were collected while the children were seated, immediately following a seated five-minute relaxation period.

### 2.3. Procedures

Anthropometric measurements (i.e., BMI and WHtR), blood markers, and blood pressure measurements were collected on the same testing day. Anthropometric measurements were collected first, blood markers collected second, and blood pressure measurements collected third for all students. Students not reporting to the data collection site in a fasting state were rescheduled.

### 2.4. Data Processing

Each child's BMI was stratified into FITNESSGRAM's HFZ or NI [22]. Only two of the three FITNESSGRAM fitness zones were used for classification in order to create a binary predictor variable for BMI. The cardiometabolic blood marker continuous variable scores were also stratified into a binary classification scheme based on US National Heart, Lung, and Blood Institute recommendations [23]. Unfavorable cardiometabolic measurements were defined as having total cholesterol ≥ 170 mg/dL, LDL cholesterol ≥ 110 mg/dL, HDL cholesterol ≤ 45 mg/dL, triglycerides ≥ 90 mg/dL, blood glucose ≥ 100 mg/dL, and systolic and diastolic blood pressure measurements ≥ 95th percentile as determined by age and sex. The aforementioned binary variables were coded as 0 = not meeting recommendations and 1 = meeting recommendations for WHtR and blood markers and 0 = NI and 1 = HFZ for BMI.

### 2.5. Statistical Analysis

For descriptive purposes, differences between sex groups on all continuous measures were examined using independent *t*-tests. The primary analysis involved using a Receiver Operating Characteristic (ROC) curve to determine the optimal WHtR cut-point needed to accurately discriminate children who did and who did not achieve FITNESSGRAM's age- and sex-specific recommendations for BMI. Overall diagnostic power was determined using the area-under-the-curve (AUC). AUC scores of ≥0.90 were considered excellent; 0.80–0.89, good; 0.70–0.79, fair; and <0.70, poor [21]. The optimal WHtR cut-point was determined using maximum Youden's *J* statistic (*J*_max_), which was calculated using STATA's “senspec” command. Youden's *J* is the point on the ROC curve that maximizes the sum of sensitivity and specificity (*J*_max_ = max((sensitivity + specificity) − 1)). Sensitivity was the probability that a child achieved a WHtR cut-point (T^+^) given that he or she met the FITNESSGRAM standard for BMI (D^+^), or P (T^+^∣D^+^). Sensitivity is synonymous with the probability of achieving a true positive. Specificity was the probability that a child did not meet a WHtR cut-point (T^−^) given that he or she did not meet the FITNESSGRAM standard for BMI, P (T^−^∣D^−^), or a true negative [24]. Maximizing sensitivity and specificity associates with the datum closest to (0,1) on the ROC curve and is a WHtR cut-point that is likely to yield strong classification accuracy.

Classification agreement between children meeting the WHtR cut-point and children meeting each cardiometabolic blood marker standard was examined using kappa statistics and percentage of agreement. The kappa statistics were interpreted as weak if <0.20, fair if 0.20–0.39, moderate if 0.40–0.59, good if 0.60–0.79, and very good if ≥0.80 [25]. Alpha level was set at *p* ≤ 0.05 and all analyses were carried out using STATA v14.0 statistical software package (College Station, TX, USA).

## 3. Results

The descriptive statistics for all continuous variables are presented in Table 1 for the total sample and within sex groups. Comparing sex groups, girls displayed higher triglycerides than boys (mean difference = 12.4 mg/dL, *p* < 0.01) and boys displayed higher diastolic blood pressure than girls (mean difference = 3.6 mmHg, *p* < 0.01). There were no other statistically significant differences between sexes for any other measure.  Table 2 presents the distribution of children meeting cardiometabolic blood marker recommendations from the National Heart, Lung, and Blood Institute. The range for meeting the various recommendations was 51.5% for triglycerides to 91.7% for blood glucose.

**Figure 1:**
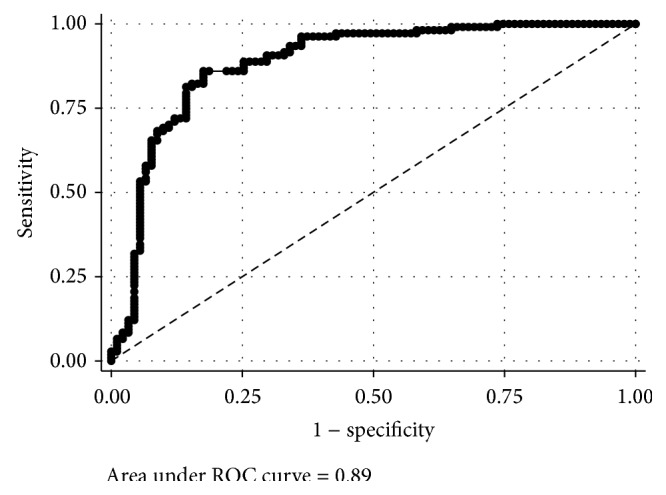
Receiver operating characteristic curve displaying the sensitivity and 1 − specificity scores for waist-to-height ratio cut-points associating with children meeting FITNESSGRAM's body composition criterion-referenced standards.

Figure 1 is the ROC curve showing the range of sensitivity and 1 − specificity for various WHtR cut-points associating with a child meeting FITNESSGRAM's age- and sex-specific criterion-referenced standards for BMI. Results from the ROC curve analysis yielded an optimal WHtR cut-point of 0.50 (*J*_max_ = 0.68, AUC = 0.89, and *p* < 0.001; sensitivity = 0.86, specificity = 0.82, and accuracy = 84.3%). The AUC was considered good. Using the derived cut-point, approximately 55% of the sample displayed a WHtR ≤ 0.50 (120/219).  Table 3 presents the agreement statistics between children meeting the derived WHtR cut-point with children meeting the recommendations for each cardiometabolic blood marker. All kappa statistics were statistically significant except for LDL cholesterol. Statistically significant kappa statistics were considered weak to fair and ranged from kappa = 0.14 for total cholesterol to kappa = 0.34 for systolic blood pressure. The percentage agreement thus ranged from 58.6% for total cholesterol to 66.8% for systolic blood pressure.

## 4. Discussion

The purpose of this study was to establish a WHtR cut-point that associated with FITNESSGRAM's age- and sex-specific criterion-reference standards for BMI. A secondary aim was to use the cut-point to analyze classification agreement with various cardiometabolic blood markers using recommendations from the National Heart, Lung, and Blood Institute. The primary finding from this study was that the derived WHtR cut-point of 0.50 strongly agreed with BMI criterion-referenced standards used in the FITNESSGRAM battery. Approximately 84% of children were correctly classified using the 0.50 cut-point and only 16% of children were misclassified. The 0.50 WHtR cut-point has been recommended in other works within the child and adolescent populations [24, 26, 27].

The simple recommendation of keeping a waist circumference less than one-half of height holds merit in the low-income pediatric population as well when distinguishing children who achieved FITNESSGRAM's body composition standards. In other work, this cut-point has been shown to relate moderately well to cardiometabolic risk factors in children, adolescents, and adults [28, 29]. The established WHtR cut-point has the benefit of being developed from FITNESSGRAM's criterion-referenced standards for BMI. FITNESSGRAM's criterion-referenced standards for BMI were developed from percent of body fat estimated from skinfold thickness, which was linked to the metabolic syndrome using a large sample of children and adolescents from the US National Health and Nutrition Examination Survey [11]. The BMI standards are currently used as part of a comprehensive fitness test battery in physical activity or physical education settings. Other researches have found WHtR cut-points ranging between 0.60 in obese Mexican adolescents [30], 0.47 in young Brazilian children [31], 0.465 in female and 0.455 in male South African children [32], and 0.475 in female and 0.485 in male Chinese children [18]. The discordance in developed WHtR cut-points may be the result of the referent variable used for comparison (e.g., metabolic syndrome, percent of body fat, and BMI), genetics, diet, age, sex, and the procedures and instrumentation used to collect anthropometric and health measurements [33]. Despite this, the cut-points derived from various studies approximate the 0.5 cut-point found in this study, which is in exact accordance with the weighted mean boundary found from a recent meta-analysis [33].

In addition to its relative ease of interpretation, WHtR also has the benefit of capturing visceral adipose deposition, which has been shown to increase low-grade systemic inflammation in the body, a possible genesis for early incident cardiometabolic disease risk factors [34]. One limitation of WHtR is that some administered training may be needed to yield a reliable and valid waist circumference measurement, whereas, with BMI, no training is needed. However, WHtR in many studies has been shown to classify individuals of all ages with greater accuracy than BMI because of its ability to isolate central adiposity [35]. Height and bone structure confounding is partially controlled for when dividing the waist measurement by height; therefore its validity as a body composition index is robust regardless of stature.

Although WHtR strongly agreed with BMI standards, its ability to distinguish children who did or did not meet individual cardiometabolic blood markers was classified as weak to fair. Most studies show an association between WHtR and individual and clustered cardiometabolic biomarkers [36]. WHtR has also been found to be associated with certain health behaviors in children such as TV viewing, sedentary behavior, and irregular breakfast [37]. In this study the accuracy in distinguishing children with unfavorable cardiometabolic biomarkers has been found to be similar compared to previous research [31, 32]. The relative lower accuracy of WHtR in children compared to older cohorts may be because of the greater difficulty to detect unfavorable risk factors because these traits may take several years to develop. Indeed, the prevalence for unfavorable biomarkers was less than 50% for most measures, except for HDL cholesterol and diastolic blood pressure, and quite low for total cholesterol, LDL cholesterol, and fasting glucose. Another reason for the weak accuracy in WHtR classifying children may have been because of the use of a capillary blood sample to collect the biomarkers. Capillary blood sampling, although convenient, may overestimate certain biomarkers compared to venous blood sampling [38]. Future research should use venous blood sampling to possibly yield more valid results.

This is the first study to establish a WHtR cut-point associating with FITNESSGRAM's criterion-references standards for BMI in low-income children. This is also the first study to associate WHtR with standards developed from FITNESSGRAM. Practically, the results from this study yield certain implications. WHtR can be used as an alternative to BMI in field settings, specifically in physical education settings for body composition assessment. As stated previously, WHtR has the benefit of capturing central adipose deposits that pose more of a health threat compared to subcutaneous deposits on a child's body [30]. Screening for central adiposity at an early age may help attenuate the risk of developing visceral adipose deposits later in life and thus attenuate incidence of unfavorable cardiometabolic disease risk factors. Although the accuracy of distinguishing low-income children with unfavorable cardiometabolic disease risk was weak to fair, statistically significant agreement was still found; therefore there is still utility for WHtR's use as a cardiometabolic screening tool. However, future research needs to explore these associations further using larger sample sizes, venous blood sampling, and more ethnically diverse samples.

There are limitations to this study that must be considered before generalizations can be made. First, the sample consisted of low-income children, primarily Hispanic, from schools located within the Mountain West region of the USA. Therefore, the external validity of the results is questionable if the results are to be generalized to higher socioeconomic status children or to samples comprising different ethnic representation. Second, a capillary blood sample was obtained to analyze the cardiometabolic blood markers, which may overestimate levels within 5% compared to venous blood samples. Third, the acquired sample size of 219 children is relatively low for cross-sectional descriptive studies employing ROC curves analysis; therefore future research should address the research question using larger sample sizes to improve statistical power and the internal validity of the results. Finally, diet was not accounted for in the analysis, which may influence cardiometabolic health markers. Future research should account for the potential confounding of diet when examining the relationship between WHtR and cardiometabolic health.

## 5. Conclusion

In conclusion, the derived WHtR cut-point of 0.50 strongly agreed with FITNESSGRAM's criterion-referenced standards for BMI. The WHtR = 0.50 cut-point has also been indicated in prior research within the adolescent and adult populations and may provide a valid alternative to BMI as a body composition assessment metric in low-income children. Despite the strong agreement with BMI, its ability to distinguish low-income children who achieved individual blood marker recommendations was weak to fair. This may have been because of the low prevalence of certain risk factors or the use of capillary blood sampling. This study was the first to establish a WHtR cut-point that associated with criterion-referenced BMI using a sample of low-income children from the USA. The WHtR = 0.50 cut-point can be used with strong accuracy in field settings to distinguish children who would meet standards using FITNESSGRAM's BMI, but its use for screening for a child's cardiometabolic disease risk needs further exploration.

## Figures and Tables

**Table 1:** Descriptive data for the total sample and within sex groups (means and standard deviations).

	Total sample (*N* = 219)	Girls (*n* = 126)	Boys (*n* = 93)
BMI^a^ (kg/m^2^)	19.1 (4.3)	18.9 (4.6)	19.3 (3.8)
WHtR^b^	0.49 (0.08)	0.48 (0.08)	0.50 (0.07)
Total cholesterol (mg/dL)	153.1 (27.7)	153.1 (26.4)	153.2 (29.4)
LDL cholesterol (mg/dL)	87.1 (26.7)	85.3 (24.4)	90.0 (29.9)
HDL cholesterol (mg/dL)	44.6 (13.2)	44.5 (12.1)	44.7 (14.6)
Triglycerides (mg/dL)	112.6 (82.8)	**112.0** ^†^ ** (94.1)**	99.6 (62.2)
Glucose (mg/dL)	86.1 (9.6)	86.1 (10.0)	86.1 (9.0)
Systolic blood pressure (mmHg)	112.9 (14.6)	113.0 (14.4)	112.9 (15.0)
Diastolic blood pressure (mmHg)	67.1 (10.9)	65.6 (10.5)	**69.2** ^†^ ** (11.2)**

*Note*. ^a^BMI stands for body mass index; ^b^WHtR stands for waist-to-height ratio; bold indicates statistical differences compared to the opposite sex, ^†^ *p* < 0.01.

**Table 2:** Number of children meeting standards/recommendations for each measure (expressed as counts and percentages).

	Meeting	% meeting	Not meeting
BMI	118	53.8%	101
Total cholesterol	172	78.5%	47
LDL cholesterol	193	88.1%	26
HDL cholesterol	98	44.7%	121
Triglycerides	113	51.5%	106
Glucose	201	91.7%	18
Systolic blood pressure	107	48.8%	112
Diastolic blood pressure	138	63.0%	81

**Table 3:** Classification agreement using the derived WHtR cut-point and cardiometabolic blood marker recommendations.

	Kappa (95% CI)	*p* value	% of agreement
Total cholesterol	**0.14 (0.01, 0.26)**	0.015	58.6%
LDL cholesterol	0.04 (−0.07, 0.16)	0.215	53.1%
HDL cholesterol	**0.33 (0.20, 0.46)**	<0.001	66.5%
Triglycerides	**0.22 (0.08, 0.36)**	0.001	61.5%
Glucose	**0.13 (0.04, 0.21)**	0.002	59.1%
Systolic blood pressure	**0.34 (0.21, 0.48)**	<0.001	66.8%
Diastolic blood pressure	**0.26 (0.13, 0.39)**	<0.001	64.0%
